# Glucocorticoid Receptor (GR) Expression in Human Tumors: A Tissue Microarray Study on More than 14,000 Tumors

**DOI:** 10.3390/biomedicines13071683

**Published:** 2025-07-09

**Authors:** Maria Christina Tsourlakis, Simon Kind, Sebastian Dwertmann Rico, Sören Weidemann, Katharina Möller, Ria Schlichter, Martina Kluth, Claudia Hube-Magg, Christian Bernreuther, Guido Sauter, Andreas H. Marx, Ronald Simon, Ahmed Abdulwahab Bawahab, Florian Lutz, Viktor Reiswich, Davin Dum, Stefan Steurer, Eike Burandt, Till S. Clauditz, Till Krech, Christoph Fraune, Seyma Büyücek, Neele Heckmann, Natalia Gorbokon, Maximilian Lennartz, Sarah Minner, Florian Viehweger

**Affiliations:** 1 Institute of Pathology, University Medical Center Hamburg-Eppendorf, 20246 Hamburg, Germany; mtsourlakis@uke.de (M.C.T.); s.kind@uke.de (S.K.); s.dwertmann-rico@uke.de (S.D.R.); s.weidemann@uke.de (S.W.); ka.moeller@uke.de (K.M.); r.schlichter@uke.de (R.S.); m.kluth@uke.de (M.K.); c.hube@uke.de (C.H.-M.); c.bernreuther@uke.de (C.B.); g.sauter@uke.de (G.S.); f.lutz@uke.de (F.L.); v.reiswich@uke.de (V.R.); d.dum@uke.de (D.D.); s.steurer@uke.de (S.S.); e.burandt@uke.de (E.B.); t.clauditz@uke.de (T.S.C.); t.krech@uke.de (T.K.); c.fraune@uke.de (C.F.); s.bueyuecek@uke.de (S.B.); n.heckmann@uke.de (N.H.); n.gorbokon@uke.de (N.G.); m.lennartz@uke.de (M.L.); s.minner@uke.de (S.M.); f.viehweger@uke.de (F.V.); 2 Department of Pathology, Academic Hospital Fuerth, 90766 Fuerth, Germany; andreas.marx@klinikum-fuerth.de; 3 Department of Basic Medical Sciences, Pathology Division, College of Medicine, University of Jeddah, Jeddah 23890, Saudi Arabia; bawahab2002@hotmail.com; 4 Institute of Pathology, Clinical Center Osnabrueck, 49076 Osnabrueck, Germany

**Keywords:** glucocorticoid receptor, immunohistochemistry, tissue microarray, cancers, prognosis

## Abstract

**Background:** The glucocorticoid receptor (GR) regulates the transcription of thousands of genes. In cancer, both oncogenic and tumor suppressive roles of GR have been proposed. **Methods**: A tissue microarray containing 18,527 samples from 147 tumor (sub-)types and 608 samples from 76 normal tissue types was analyzed for GR expression by immunohistochemistry. **Results**: GR positivity was found in 76.4% of 14,349 interpretable cancers, including 18.5% with weak, 19.6% with moderate, and 38.3% with strong positivity. GR positivity appeared in all 147 tumor types, with at least one strongly positive tumor in 136 types. Of out tumor entities, 77 of the 147 showed GR positivity in 100% of the cases analyzed. Only six tumor types had less than 50% GR-positive cases, including adenomas with low-/high-grade dysplasia (32.5%/21.7%), adenocarcinomas (17%) and neuroendocrine carcinomas (45.5%) of the colorectum, endometrial carcinomas (25.6%), and rhabdoid tumors (25%). Reduced GR staining was associated with grade progression in pTa (*p* < 0.0001) and with nodal metastasis in pT2-4 (*p* = 0.0051) urothelial bladder carcinoma, advanced pT stage (*p* = 0.0006) in breast carcinomas of no special type (NST), and high grade (*p* = 0.0066), advanced pT stage (*p* < 0.0001), and distant metastasis (*p* = 0.0081) in clear cell renal cell carcinoma. GR expression was unrelated to clinico-pathological parameters in gastric, pancreatic, and colorectal adenocarcinoma, and in serous high-grade carcinoma of the ovary. **Conclusions**: GR expression is frequent across all cancer types. Associations between reduced GR expression and unfavorable tumor features in certain cancers suggest that the functional importance of GR-regulated genes in cancer progression depends on the cell of tumor origin.

## 1. Introduction

The glucocorticoid receptor (GR) is a multidomain nuclear protein which is coded by the *NR3C1* (nuclear receptor subfamily 3, group C, member 1) gene at 5q31 [1,2]. GR is one of the members of the nuclear receptor (NR) superfamily subgroup 3 which also contains the androgen receptor (AR), mineralocorticoid receptor (MR), progesterone receptor (PR) and the two estrogen receptors (ERα and ERβ) (reviewed in [3]). GR is the most relevant receptor protein for cortisol and other glucocorticoids [4]. Depending on ligand binding and the recruitment of context-specific transcriptional coregulators, GR modulates either the activation or repression of the transcription of a broad range of different genes involved in development, metabolism, stress, and inflammatory responses (reviewed in [5,6]). GR and glucocorticoids have a critical impact on diverse physiological processes (reviewed in [5]) and are integral to the treatment of hematological malignancies due to their potent anti-inflammatory and lympholytic effects [7].

Multiple studies have provided evidence that GR expression plays a complex role in tumorigenesis which goes way beyond its effects on the immune system. Both oncogenic and tumor-suppressive roles of GR have been found depending on tumor type and on specific tumor characteristics (reviewed in [8]). For example, in breast cancer, GR expression was linked to favorable patient outcomes in ER-positive cancer [9,10] but related to shorter breast cancer specific survival, poor prognosis, resistance to chemotherapy, and metastasis in ER-negative cancer [9,11,12]. In prostate cancer, GR activation inhibited tumor angiogenesis [13] and reduced proliferation in primary hormone-sensitive prostate cancer cell lines [14], while GR signaling facilitated resistance to anti-androgen therapies in advanced prostate cancer [15,16]. Most of the clinical interest in GR expression in cancer comes from its potential as a druggable target, although the effects of glucocorticoid treatment in solid cancers are complex and sometimes controversial. For example, GR antagonists have shown promise in the therapy of triple negative breast cancer [12], but the effects of glucocorticoid therapies in hormone receptor positive breast cancer can vary with its subtype and molecular profile [17]. In prostate cancer, GR overexpression has been shown to constitute a mechanism for androgen resistance [15,18]. In concordance with these findings, preclinical studies provided evidence that inhibition of the GR pathway leads to re-sensitization to antiandrogen therapy and chemotherapeutics like docetaxel [16,19].

Despite of its potential importance, data on GR protein expression in tumors are still sparse and considerably variable. For example, the reported range of GR positivity found in studies using immunohistochemistry (IHC) for GR detection ranged from 0 to 100% in breast carcinoma [11,20,21,22,23,24,25,26,27,28,29,30,31,32,33,34,35,36], 20–100% in prostate carcinoma [14,24,37,38,39], 27–100% in ductal adenocarcinoma of the pancreas [24,40,41,42], 45–94% in non-small cell lung cancer [43,44,45,46,47], and 0–48% in colorectal adenocarcinoma [41,48]. Varying positivity rates in different IHC studies are likely due to the use of different antibodies, staining protocols, and criteria for defining positivity.

To overcome such shortcomings, a comprehensive and highly standardized study using one highly validated antibody and immunohistochemistry protocol to analyze many tumors from different tumor entities is of interest. Accordingly, GR expression was analyzed in more than 14,000 tumor tissue samples from 147 different tumor types and subtypes as well as 76 different non-neoplastic tissue types by IHC in a tissue microarray (TMA) format in this study.

## 2. Materials and Methods

### 2.1. Tissue Microarrays (TMAs)

Our normal TMA was composed of 8 samples from 8 different donors of 76 different normal tissue types (608 samples on one slide). The cancer TMAs included a total of 18,527 primary tumors from 147 different tumor types and subtypes. Detailed histopathological data were available for invasive breast carcinomas of no special type (n = 1680), urothelial carcinomas of the bladder (n = 2434), colorectal adenocarcinomas (n = 2351), endometrioid endometrial carcinomas (n = 182), clear cell renal cell carcinomas (n = 1224), papillary renal cell carcinomas (n = 310), serous high-grade ovarian carcinomas (n = 369), ductal adenocarcinomas of the pancreas (n = 598), papillary thyroid carcinomas (n = 382), gastric adenocarcinomas (n = 327), and germ cell tumors of the testis (n = 565). Clinical follow up data were accessible for 717 patients with invasive breast carcinoma of no special type (NST) patients with a median follow-up time of 50 months. The composition of normal and cancer TMAs is described in the Section 3. All samples were from the archives of the Institute of Pathology, University Hospital of Hamburg, Germany, the Institute of Pathology, Clinical Center Osnabrueck, Germany, and the Department of Pathology, Academic Hospital Fuerth, Germany. The samples were collected from patients who underwent surgery for tumor resection between 1994 and 2020. Data on patient gender were available for 10,398 tumor samples with interpretable GR data and included 4537 males and 5861 females. Tissues were fixed in 4% buffered formalin and then embedded in paraffin. The TMA manufacturing process was described previously in detail [49]. In brief, one tissue spot (diameter: 0.6 mm) per tissue sample (one sample per patient) patient was used. The use of archived remnants of diagnostic tissues for TMA manufacturing, their analysis for research purposes, and the use of patient data were carried out according to local laws (HmbKHG, §12) and our analysis was approved by the local ethics committee (Ethics commission Hamburg, WF-049/09). All work has been carried out in compliance with the Helsinki Declaration.

### 2.2. Immunohistochemistry (IHC)

Freshly cut TMA sections were immunostained on one day and in one experiment. Slides were deparaffinized with xylol, rehydrated through a graded alcohol series, and exposed to heat-induced antigen retrieval for 5 min in an autoclave at 121 °C in pH 7.8 Tris-EDTA-Citrat (TEC) buffer. Endogenous peroxidase activity was blocked with Dako REAL Peroxidase-Blocking Solution (Agilent Technologies, Santa Clara, CA, USA; #S2023) for 10 min. A primary antibody specific for the Glucocorticoid receptor (GR) (HMV304, rabbit recombinant monoclonal, ardoci GmbH, Hamburg, Germany) was applied at 37 °C for 60 min at a dilution of 1:150. For the purpose of antibody validation, the normal tissue TMA was also analyzed using the rabbit recombinant monoclonal antibody EPR19621 (Abcam, Cambridge, MA, USA, #ab183127) at a dilution of 1:600 and an otherwise identical protocol. The bound antibody was then visualized using the Dako REAL EnVision Detection System Peroxidase/DAB+, Rabbit/Mouse kit (Agilent Technologies, Santa Clara, CA, USA; #K5007) according to the manufacturer’s directions. The sections were counterstained with hemalaun. For tumor tissues, all tumor cells present in a given tissue spot were scored. The percentage of positive neoplastic cells was estimated, and the staining intensity was semi-quantitatively recorded (0, 1+, 2+, 3+). For statistical analyses, the staining results were categorized into four groups. Tumors without any staining were considered negative. Tumors with 1+ staining intensity in ≤70% of tumor cells or 2+ intensity in ≤30% of tumor cells were considered weakly positive. Tumors with 1+ staining intensity in >70% of tumor cells, 2+ intensity in 31–70%, or 3+ intensity in ≤30% of tumor cells were considered moderately positive. Tumors with 2+ intensity in >70% or 3+ intensity in >30% of tumor cells were considered strongly positive.

### 2.3. Statistics

Statistical calculations were performed with JMP17^®^ software (SAS^®^, Cary, NC, USA). Levene’s test for normality and homogeneity was used to assess similar variances before statistical analysis. Contingency tables and the chi^2^-test were performed to search for associations between GR immunostaining and tumor phenotype. The Log-Rank test was applied to detect significant differences between groups. A *p*-value of ≤0.05 was defined as significant.

## 3. Results

### 3.1. Technical Issues

A total of 14,349 (77.4%) of the 18,527 tumor samples collected were interpretable in our TMA analysis. The non-interpretable samples demonstrated a lack of unequivocal tumor cells or a lack of entire tissue spots. A sufficient number of samples (≥4) of each normal tissue type was evaluable.

### 3.2. Glucocorticoid Receptor Immunostaining in Normal Tissues

**Figure 1:**
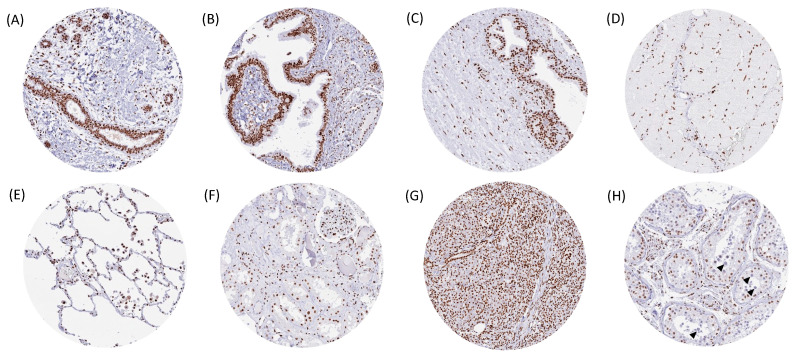
GR immunostaining of normal tissues. The panels show a variable but usually strong nuclear GR in all cell types of the breast (**A**), fallopian tube (**B**), prostate (**C**), skeletal muscle (**D**), lung (**E**), kidney (**F**), and the pancreas, (**G**) while GR staining is markedly reduced in cells of spermiogenesis in the testis (see arrowheads in (**H**)).

GR immunostaining was always nuclear and seen in almost all cell types of all organs. Cell types with either reduced or absent GR staining included the upper cell layers of the non-keratinizing squamous epithelium and of the urothelium, corpuscles of Hassall’s from the thymus, glandular cells from the stomach, crypt base epithelial cells from the appendix and the colorectum, glandular cells from the endometrium in the secretion phase, cells from the spermiogenesis, granulosa cells from the ovary, adrenocortical cells, and trophoblastic cells from the mature (and not the first trimenon) placenta. Tissues with at least moderate-to-strong GR staining of all cell types or cell types with at least moderate GR staining included the prostate, seminal vesicle, epididymis, Sertoli and Leydig cells from the testis, fallopian tube, endocervix, stroma cells from the endometrium, myometrium, theca interna, corpus luteum, and stromal cells from the ovary, breast, basal respiratory epithelium, skeletal muscle cells, smooth muscle cells, kidney, gallbladder, pancreas (stronger staining in islet cells than in acinar cells), salivary glands, thyroid, decidua cells and endometrium of the pregnant uterus, cells of the aortic wall, medullary cells of the adrenal gland, parathyroid, hematopoietic and lymphoid tissues, lung, hypophysis, and neurons from the cerebrum. Examples of GR staining in normal tissues are shown in Figure 1. All these staining patterns were observed by using both HMV304 and EPR19621 (Supplementary Figure S1).

### 3.3. Glucocorticoid Receptor Immunostaining in Neoplastic Tissues

GR staining was always nuclear in the tumors. GR positivity was found in 76.4% of the 14,349 interpretable cancers, including 18.5% with weak, 19.6% with moderate, and 38.3% with strong positivity. GR positivity of at least a fraction of tumors was found in all 147 tumor types analyzed and at least one strongly GR-positive tumor was found in 136 tumor types (Table 1).

**Figure 2:**
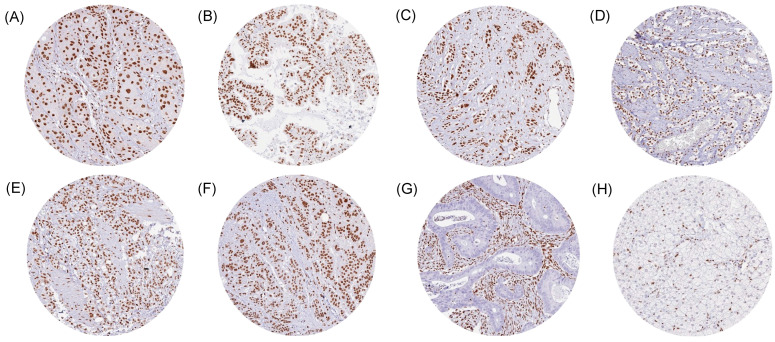
GR immunostaining in cancer. The panels show a moderate-to-strong GR positivity in tumor cells from squamous cell carcinoma of the cervix (**A**), adenocarcinoma of the lung (**B**), ductal adenocarcinoma of the pancreas (**C**), prostatic adenocarcinoma (**D**), gastric adenocarcinoma (**E**), and adrenocortical carcinoma (**F**). GR staining is lacking in tumor cells from a colorectal adenocarcinoma (**G**) and an adrenocortical adenoma (**H**) while distinct GR staining occurs in stromal cells.

Of our tumor entities, 77 of the 147 showed GR positivity of variable intensity in all analyzed cases. A further 43 tumor entities showed GR positivity in 80–99.9% of cases. And 21 tumor entities had a GR positivity rate between 50 and 80%, including urothelial carcinoma as well as gastric and esophageal adenocarcinoma. Only six tumor types had less than 50% GR-positive samples. These included adenomas with low- and high-grade dysplasia (32.5% and 21.7%), adenocarcinomas (17%) and neuroendocrine carcinomas (45.5%) of the colorectum, and endometrioid endometrial carcinomas (25.6%), as well as rhabdoid tumors (25%). Representative images of GR-positive and -negative tumors are shown in Figure 2.

**Figure 3:**
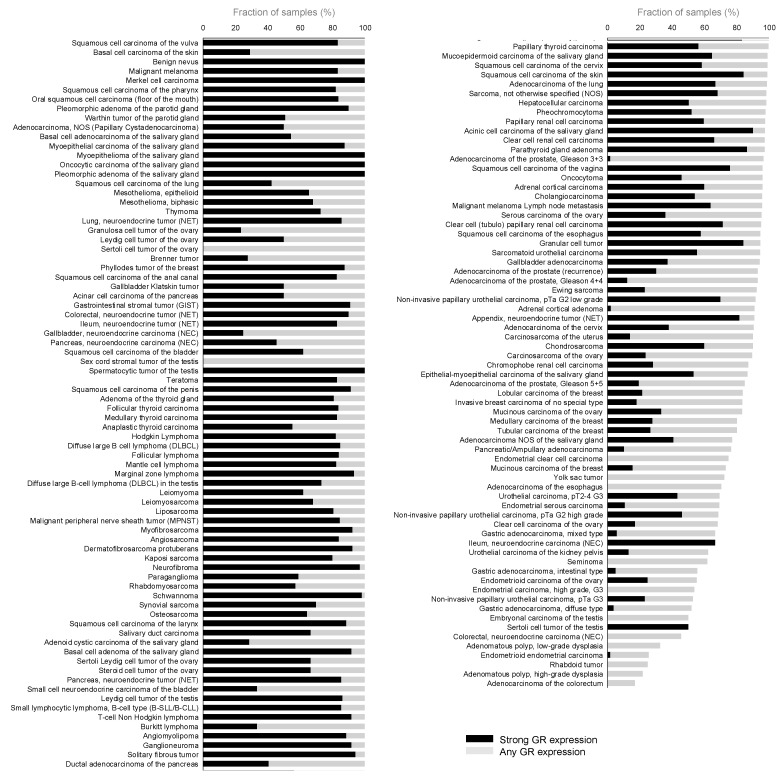
Ranking order of GR immunostaining in tumors. Both the overall percentage of positive cases (gray bars) and the fraction of strongly positive cases (black bars) are shown.

A ranking of tumor categories according to the rate of GR positivity is given in Figure 3.

Reduced GR staining was significantly associated with adverse histopathological and clinical features in multiple tumor types (Table 2).

Low GR expression was linked to grade progression in pTa (*p* < 0.0001) and to nodal metastasis in pT2-4 (*p* = 0.0051) urothelial carcinoma of the urinary bladder and advanced pT stage (*p* = 0.0006) in breast carcinomas of no special type (NST), as well as to high-grade (*p* = 0.0066), advanced pT stage (*p* < 0.0001), and distant metastasis (*p* = 0.0081) in clear cell renal cell carcinoma. GR expression was unrelated to clinico-pathological parameters in gastric, pancreatic and colorectal adenocarcinoma, as well as in serous high-grade carcinoma of the ovary.

## 4. Discussion

Our successful evaluation of more than 14,000 samples from 147 different tumor types and subtypes provides a comprehensive overview on the prevalence of GR expression in cancer. That GR expression was observed in all 147 cancer entities and that only six of them showed a positivity rate below 50% identifies GR expression as a common feature of cancers cells. In general, this finding is in line with the previous literature on GR immunostaining in cancer (summarized in Supplementary Figure S2 [20,21,22,24,26,27,29,30,31,34,35,36,40,46,47,50,51,52,53,54,55,56,57,58] although published data were controversial for several entities. The particularly low rate of GR positivity in colorectal cancer (17.1%, including 14.5% with low level positivity) is in agreement with an earlier study reporting negative GR immunostaining in all 35 analyzed colorectal adenocarcinomas [41]. The frequent high-level GR positivity in the vast majority of cancer types indicates limited potential for GR IHC as a tool for the distinction of tumor entities. There are, however, two possible applications which may deserve further evaluation. The much higher GR positivity rate in adrenocortical carcinoma (88% of 25 with moderate/strong staining) than in adrenocortical adenoma (15.5% of 45 with moderate/strong staining) suggests that GR IHC could help in the otherwise challenging distinction of benign from malignant adrenocortical neoplasms (reviewed in [59]). Based on the very low frequency of colorectal adenocarcinomas with moderate (2.4%) and strong (0.2%) GR positivity, a significant GR positivity by IHC would argue against a colorectal origin of an adenocarcinoma metastasis. Other adenocarcinomas, such as those from the pancreas (84.3%), the gallbladder (77.1%), the stomach (24.4%), the esophagus (36.4%), the lung (92.1%), or the prostate (56.7%) had markedly higher rates of moderate-to-strong GR positivity.

The availability of large tumor cohorts from several frequent cancer types enabled us to evaluate the relationship between GR expression and clinico-pathological parameters of cancer aggressiveness in several tumor types. That reduced GR expression was linked to unfavorable tumor features in breast cancer, clear cell renal cell carcinoma, and in urothelial carcinoma is in line with data from earlier studies [11,25,60,61]. Associations between reduced GR expression and unfavorable tumor features have also been described for adrenocortical carcinomas [50], thymic epithelial tumors [62], ductal adenocarcinoma of the pancreas [42], cervical cancer [56], colorectal adenocarcinomas, and non-small cell lung cancer [45]. Overall, these observations argue for a tumor-suppressive role of GR in these tumor entities. Data from functional studies have supported the tumor suppressive function of GR in some tumor types. Caratti et al. found that deletion of the GR in A549 lung cancer cells enhanced tumor growth of xenografts in mice [63]. Matthews et al. reported an essential role of GR for proper cell cycle progression in HeLa cells and observed mitotic aberrations and tumor formation in mice that were haploinsufficient for GR [64]. Yemelyanov et al. described an inhibition of cell proliferation and a blockage of anchorage-independent growth after lentiviral reconstitution of GR expression in GR-negative LNCaP prostate cancer cells [14]. Alternatively, reduced GR expression in tumors derived from GR expressing normal cells could just reflect tumor cell dedifferentiation paralleling cancer progression. Importantly, low GR expression is not a general feature of poor prognosis in cancer. GR expression was unrelated to clinico-pathological features in multiple tumor entities in this study. Moreover, a strong and independent correlation between high GR expression and poor clinical outcomes was recently found by our group in a cohort of 12,152 prostate cancer samples by using the same IHC assay as in this study (Heckmann et al., submitted). Others have also found a link between high GR expression and unfavorable tumor phenotype in prostate cancer [65] as well as in several other cancer types such as ovarian cancer [51,66], endometrial cancer [57], salivary duct carcinoma [52], malignant melanoma [67], and squamous cell carcinoma of the esophagus [53]. The mechanisms by which GR upregulation was found to increase cancer aggressiveness include promotion of epithelial–mesenchymal transformation by transcriptional repression of insulin receptor substrate 1 in breast cancer cells [68], escape from apoptosis by repression of p38 MAP kinase in cervical cancer cells [69], bypass of androgen receptor blockade in prostate cancer cells [70], and increased glycolytic energy production through the suppression of mitochondrial pyruvate dehydrogenase in liver cancer cells [71].

Glucocorticoids are widely used in cancer patients to reduce the side effects of chemotherapy and radiation and to protect healthy tissue from toxicity. Considering the widespread but variable expression of GR in cancer, it must be assumed that the application of steroids can also directly impact cancer cells and exert clinical effects that may remain clinically unrecognized in the context of severe illness in affected patients. Several studies have indeed demonstrated the direct effects of steroid hormones on tumor cell behavior. The GR agonist dexamethasone induced a gene signature that correlated with shorter survival in The Cancer Genome Atlas glioblastoma dataset [72], enhanced tumor growth and metastasis in breast cancer xenograft mouse models [73], transcriptionally activated TEA domain transcription factor 4, the expression of which correlates with poor survival of patients with breast cancer [74], and favored epithelial–mesenchymal transition, self-renewal potential, and cancer progression in pancreatic ductal adenocarcinoma cell lines [75]. Although GR antagonists are generally explored for their potential to mitigate the pro-tumorigenic effects of glucocorticoids, there is some evidence that also antagonists such as mifepristone may promote tumor progression in specific settings. For example, mifepristone significantly stimulated ovarian cancer cell migration, proliferation, and growth in vivo [76], and promoted testicular Leydig cell tumor progression in transgenic mice [77]. Other studies have demonstrated that GR agonists and antagonists can impact tumor cell responses to chemotherapy in several experimental models of solid tumors. For example, GR agonists induced chemotherapy resistance in lung cancer, prostate cancer, breast cancer and ovarian cancer cells [15,78,79,80,81]. GR antagonists, such as mifepristone or relacorilant could reverse chemotherapy resistance and potentiate chemotherapy-induced apoptosis in triple-negative breast cancer, pancreatic cancer, and ovarian cancer cell lines [12,82].

Given the large scale of our study, particular emphasis was placed on a thorough validation of our assay. The International Working Group for Antibody Validation (IWGAV) has proposed that an acceptable antibody validation for immunohistochemistry on formalin fixed tissues must include either a comparison of IHC findings with a second antibody for the same target or a comparison with another independent method for expression analysis [83]. Because comparison with a method using disaggregated tissue is not well suited in case of ubiquitously expressed proteins, we performed an extensive comparison of antibodies on 76 different categories of normal tissues. These experiments confirmed the specificity of our assay because all staining patterns obtained by HMV304 were confirmed by EPR19621. These included a reduction or loss of GR expression in upper cell layers of non-keratinizing squamous epithelium and of the urothelium, cells of the spermiogenesis, granulosa cells of the ovary, adrenocortical cells, and trophoblastic cells of the mature but not of the first trimenon placenta. It is of note that the use of a very broad range of different tissues (76 different normal tissue categories) for antibody validation increases the likelihood of detecting undesired cross-reactivities because virtually all proteins occurring in normal cells of adult humans are subjected to the validation experiment.

## 5. Conclusions

Our data provide a comprehensive overview of GR expression in normal and neoplastic human tissues. Significant associations between reduced GR expression and unfavorable tumor features in some but not all tumor types demonstrates that the functional effects of GR expression significantly depend on the tumor cell type. The potential role of GR expression as a predictive marker for both the response to GR-targeted therapies and the side-effects of GR-agonists administered to cancer patients has to be evaluated further.

## Figures and Tables

**Table 1:** GR immunostaining in human tumors.

			GR Immunostaining				
Tumor Category	Tumor Entity	On TMA (n)	Analyzable (n)	Negative (%)	Weak (%)	Moderate (%)	Strong (%)
Tumors of the skin	Basal cell carcinoma of the skin	89	62	0.0	32.3	38.7	29.0
	Benign nevus	29	20	0.0	0.0	0.0	100.0
	Squamous cell carcinoma of the skin	145	102	1.0	2.9	11.8	84.3
	Malignant melanoma	65	48	0.0	8.3	8.3	83.3
	Malignant melanoma lymph node metastasis	86	72	4.2	13.9	18.1	63.9
	Merkel cell carcinoma	2	1	0.0	0.0	0.0	100.0
Tumors of the head and neck	Squamous cell carcinoma of the larynx	109	79	0.0	2.5	8.9	88.6
	Squamous cell carcinoma of the pharynx	60	56	0.0	5.4	12.5	82.1
	Oral squamous cell carcinoma (floor of the mouth)	130	111	0.0	1.8	14.4	83.8
	Pleomorphic adenoma of the parotid gland	50	30	0.0	0.0	10.0	90.0
	Warthin tumor of the parotid gland	104	63	0.0	6.3	42.9	50.8
	Adenocarcinoma, NOS (Papillary Cystadenocarcinoma)	14	4	0.0	0.0	50.0	50.0
	Salivary duct carcinoma	15	3	0.0	0.0	33.3	66.7
	Acinic cell carcinoma of the salivary gland	181	41	2.4	0.0	7.3	90.2
	Adenocarcinoma NOS of the salivary gland	109	22	22.7	9.1	27.3	40.9
	Adenoid cystic carcinoma of the salivary gland	180	14	0.0	28.6	42.9	28.6
	Basal cell adenocarcinoma of the salivary gland	25	11	0.0	9.1	36.4	54.5
	Basal cell adenoma of the salivary gland	101	24	0.0	0.0	8.3	91.7
	Epithelial-myoepithelial carcinoma of the salivary gland	53	15	13.3	0.0	33.3	53.3
	Mucoepidermoid carcinoma of the salivary gland	343	122	0.8	18.9	15.6	64.8
	Myoepithelial carcinoma of the salivary gland	21	8	0.0	0.0	12.5	87.5
	Myoepithelioma of the salivary gland	11	4	0.0	0.0	0.0	100.0
	Oncocytic carcinoma of the salivary gland	12	2	0.0	0.0	0.0	100.0
	Pleomorphic adenoma of the salivary gland	53	17	0.0	0.0	0.0	100.0
Tumors of the lung, pleura, and thymus	Adenocarcinoma of the lung	196	151	1.3	6.6	25.2	66.9
	Squamous cell carcinoma of the lung	80	59	0.0	6.8	50.8	42.4
	Mesothelioma, epithelioid	40	32	0.0	6.3	28.1	65.6
	Mesothelioma, biphasic	29	22	0.0	9.1	22.7	68.2
	Thymoma	29	22	0.0	0.0	27.3	72.7
	Lung, neuroendocrine tumor (NET)	29	21	0.0	14.3	0.0	85.7
Tumors of the female genital tract	Squamous cell carcinoma of the vagina	78	50	4.0	6.0	14.0	76.0
	Squamous cell carcinoma of the vulva	157	120	0.0	1.7	15.0	83.3
	Squamous cell carcinoma of the cervix	136	113	0.9	9.7	31.0	58.4
	Adenocarcinoma of the cervix	23	21	9.5	19.0	33.3	38.1
	Endometrioid endometrial carcinoma	338	289	74.4	18.7	5.2	1.7
	Endometrial serous carcinoma	86	65	30.8	33.8	24.6	10.8
	Carcinosarcoma of the uterus	57	50	10.0	32.0	44.0	14.0
	Endometrial carcinoma, high grade, G3	13	13	46.2	23.1	30.8	0.0
	Endometrial clear cell carcinoma	9	8	25.0	37.5	37.5	0.0
	Endometrioid carcinoma of the ovary	130	96	44.8	22.9	7.3	25.0
	Serous carcinoma of the ovary	580	467	4.7	21.8	37.5	36.0
	Mucinous carcinoma of the ovary	101	72	16.7	15.3	34.7	33.3
	Clear cell carcinoma of the ovary	51	41	31.7	29.3	22.0	17.1
	Carcinosarcoma of the ovary	47	38	10.5	31.6	34.2	23.7
	Granulosa cell tumor of the ovary	44	38	0.0	26.3	50.0	23.7
	Leydig cell tumor of the ovary	4	4	0.0	25.0	25.0	50.0
	Sertoli cell tumor of the ovary	1	1	0.0	100.0	0.0	0.0
	Sertoli Leydig cell tumor of the ovary	3	3	0.0	0.0	33.3	66.7
	Steroid cell tumor of the ovary	3	3	0.0	33.3	0.0	66.7
	Brenner tumor	41	36	0.0	27.8	44.4	27.8
Tumors of the breast	Invasive breast carcinoma of no special type	1764	1489	16.4	31.8	33.7	18.1
	Lobular carcinoma of the breast	363	240	16.3	30.0	32.1	21.7
	Medullary carcinoma of the breast	34	25	20.0	28.0	24.0	28.0
	Tubular carcinoma of the breast	29	15	20.0	20.0	33.3	26.7
	Mucinous carcinoma of the breast	65	45	26.7	31.1	26.7	15.6
	Phyllodes tumor of the breast	50	40	0.0	2.5	10.0	87.5
Tumors of the digestive system	Adenomatous polyp, low-grade dysplasia	50	40	67.5	30.0	2.5	0.0
	Adenomatous polyp, high-grade dysplasia	50	46	78.3	21.7	0.0	0.0
	Adenocarcinoma of the colorectum	2483	2242	83.0	14.5	2.4	0.2
	Gastric adenocarcinoma, diffuse type	215	129	48.1	34.1	14.0	3.9
	Gastric adenocarcinoma, intestinal type	215	160	44.4	29.4	21.3	5.0
	Gastric adenocarcinoma, mixed type	62	51	33.3	31.4	29.4	5.9
	Adenocarcinoma of the esophagus	83	44	29.5	34.1	36.4	0.0
	Squamous cell carcinoma of the esophagus	76	38	5.3	7.9	28.9	57.9
	Squamous cell carcinoma of the anal canal	91	75	0.0	1.3	16.0	82.7
	Cholangiocarcinoma	58	48	4.2	12.5	29.2	54.2
	Gallbladder adenocarcinoma	51	35	5.7	17.1	40.0	37.1
	Gallbladder Klatskin tumor	42	30	0.0	16.7	33.3	50.0
	Hepatocellular carcinoma	312	289	1.7	18.0	29.8	50.5
	Ductal adenocarcinoma of the pancreas	659	454	0.2	15.4	43.8	40.5
	Pancreatic/Ampullary adenocarcinoma	98	77	23.4	35.1	31.2	10.4
	Acinar cell carcinoma of the pancreas	18	16	0.0	6.3	43.8	50.0
	Gastrointestinal stromal tumor (GIST)	62	56	0.0	1.8	7.1	91.1
	Appendix, neuroendocrine tumor (NET)	25	11	9.1	9.1	0.0	81.8
	Colorectal, neuroendocrine tumor (NET)	12	10	0.0	0.0	10.0	90.0
	Ileum, neuroendocrine tumor (NET)	53	47	0.0	0.0	17.0	83.0
	Pancreas, neuroendocrine tumor (NET)	101	89	0.0	3.4	11.2	85.4
	Colorectal, neuroendocrine carcinoma (NEC)	14	11	54.5	27.3	18.2	0.0
	Ileum, neuroendocrine carcinoma (NEC)	8	3	33.3	0.0	0.0	66.7
	Gallbladder, neuroendocrine carcinoma (NEC)	4	4	0.0	0.0	75.0	25.0
	Pancreas, neuroendocrine carcinoma (NEC)	14	11	0.0	0.0	54.5	45.5
Tumors of the urinary system	Non-invasive papillary urothelial ca., pTa G2 low grade	87	73	8.2	15.1	6.8	69.9
	Non-invasive papillary urothelial ca., pTa G2 high grade	80	67	31.3	10.4	11.9	46.3
	Non-invasive papillary urothelial carcinoma, pTa G3	126	108	47.2	18.5	11.1	23.1
	Urothelial carcinoma, pT2-4 G3	735	442	30.5	13.3	12.7	43.4
	Squamous cell carcinoma of the bladder	22	21	0.0	0.0	38.1	61.9
	Small cell neuroendocrine carcinoma of the bladder	5	3	0.0	0.0	66.7	33.3
	Sarcomatoid urothelial carcinoma	25	18	5.6	11.1	27.8	55.6
	Urothelial carcinoma of the kidney pelvis	62	53	37.7	30.2	18.9	13.2
	Clear cell renal cell carcinoma	1287	1124	2.6	12.5	18.9	66.1
	Papillary renal cell carcinoma	368	307	2.3	14.7	23.5	59.6
	Clear cell (tubulo) papillary renal cell carcinoma	26	21	4.8	19.0	4.8	71.4
	Chromophobe renal cell carcinoma	170	149	12.8	38.9	20.1	28.2
	Oncocytoma	257	200	4.0	19.5	30.5	46.0
Tumors of the male genital organs	Adenocarcinoma of the prostate, Gleason 3+3	83	59	3.4	50.8	44.1	1.7
	Adenocarcinoma of the prostate, Gleason 4+4	80	57	7.0	43.9	36.8	12.3
	Adenocarcinoma of the prostate, Gleason 5+5	85	67	14.9	34.3	31.3	19.4
	Adenocarcinoma of the prostate (recurrence)	258	129	7.0	24.8	38.0	30.2
	Seminoma	682	614	38.3	56.5	5.0	0.2
	Embryonal carcinoma of the testis	54	48	50.0	47.9	2.1	0.0
	Leydig cell tumor of the testis	31	29	0.0	0.0	13.8	86.2
	Sertoli cell tumor of the testis	2	2	50.0	0.0	0.0	50.0
	Sex cord stromal tumor of the testis	1	1	0.0	100.0	0.0	0.0
	Spermatocytic tumor of the testis	1	1	0.0	0.0	0.0	100.0
	Yolk sac tumor	53	40	27.5	70.0	2.5	0.0
	Teratoma	53	35	0.0	8.6	8.6	82.9
	Squamous cell carcinoma of the penis	92	81	0.0	0.0	8.6	91.4
Tumors of endocrine organs	Adenoma of the thyroid gland	113	89	0.0	2.2	16.9	80.9
	Papillary thyroid carcinoma	391	332	0.3	5.4	38.0	56.3
	Follicular thyroid carcinoma	154	111	0.0	2.7	13.5	83.8
	Medullary thyroid carcinoma	111	76	0.0	0.0	17.1	82.9
	Parathyroid gland adenoma	43	37	2.7	2.7	8.1	86.5
	Anaplastic thyroid carcinoma	45	38	0.0	7.9	36.8	55.3
	Adrenal cortical adenoma	48	45	8.9	75.6	13.3	2.2
	Adrenal cortical carcinoma	27	25	4.0	8.0	28.0	60.0
	Pheochromocytoma	51	46	2.2	15.2	30.4	52.2
Tumors of hematopoetic and lymphoid tissues	Hodgkin Lymphoma	103	67	0.0	6.0	11.9	82.1
	Small lymphocytic lymphoma, B-cell type (B-SLL/B-CLL)	50	48	0.0	0.0	14.6	85.4
	Diffuse large B cell lymphoma (DLBCL)	113	112	0.0	1.8	13.4	84.8
	Follicular lymphoma	88	88	0.0	2.3	13.6	84.1
	T-cell Non Hodgkin lymphoma	25	24	0.0	4.2	4.2	91.7
	Mantle cell lymphoma	18	17	0.0	0.0	17.6	82.4
	Marginal zone lymphoma	16	15	0.0	6.7	0.0	93.3
	Diffuse large B-cell lymphoma (DLBCL) in the testis	16	15	0.0	0.0	26.7	73.3
	Burkitt lymphoma	5	3	0.0	33.3	33.3	33.3
Tumors of soft tissue and bone	Granular cell tumor	23	19	5.3	5.3	5.3	84.2
	Leiomyoma	50	42	0.0	26.2	11.9	61.9
	Leiomyosarcoma	94	78	0.0	10.3	21.8	67.9
	Liposarcoma	96	77	0.0	5.2	14.3	80.5
	Malignant peripheral nerve sheath tumor (MPNST)	15	13	0.0	0.0	15.4	84.6
	Myofibrosarcoma	26	26	0.0	0.0	7.7	92.3
	Angiosarcoma	42	31	0.0	3.2	12.9	83.9
	Angiomyolipoma	91	79	0.0	1.3	10.1	88.6
	Dermatofibrosarcoma protuberans	21	13	0.0	0.0	7.7	92.3
	Ganglioneuroma	14	12	0.0	0.0	8.3	91.7
	Kaposi sarcoma	8	5	0.0	20.0	0.0	80.0
	Neurofibroma	117	98	0.0	0.0	3.1	96.9
	Sarcoma, not otherwise specified (NOS)	74	63	1.6	7.9	22.2	68.3
	Paraganglioma	41	39	0.0	5.1	35.9	59.0
	Ewing sarcoma	23	13	7.7	23.1	46.2	23.1
	Rhabdomyosarcoma	7	7	0.0	14.3	28.6	57.1
	Schwannoma	122	106	0.0	0.0	1.9	98.1
	Synovial sarcoma	12	10	0.0	10.0	20.0	70.0
	Osteosarcoma	19	14	0.0	21.4	14.3	64.3
	Chondrosarcoma	15	10	10.0	0.0	30.0	60.0
	Rhabdoid tumor	5	4	75.0	0.0	25.0	0.0
	Solitary fibrous tumor	17	17	0.0	0.0	5.9	94.1

**Table 2:** GR immunostaining and tumor phenotype.

Tumor Entity	Pathological and Molecular Parameters		GR Immunostaining				
		n	Negative (%)	Weak (%)	Moderate (%)	Strong (%)	*p*
Invasive breast carcinoma of no special type	pT1	688	13.1	29.9	34.9	22.1	0.0006
	pT2	587	17.9	32.9	33.4	15.8	
	pT3-4	116	25.9	33.6	29.3	11.2	
	G1	162	16.7	27.8	30.9	24.7	0.2624
	G2	744	16.8	30.4	34.1	18.7	
	G3	521	14.4	34.2	34.5	16.9	
	pN0	640	16.6	29.5	34.4	19.5	0.1622
	pN+	485	15.9	35.7	31.8	16.7	
	pM0	181	13.3	29.8	37.6	19.3	0.455
	pM1	106	19.8	31.1	33.0	16.0	
	HER2 negative	797	15.8	29.4	34.0	20.8	0.5939
	HER2 positive	118	16.9	34.7	30.5	17.8	
	ER negative	193	13.0	34.7	38.9	13.5	0.0126
	ER positive	682	17.2	28.9	32.3	21.7	
	PR negative	367	14.4	32.4	35.1	18.0	0.3353
	PR positive	541	16.3	28.5	33.5	21.8	
	non-triple negative	722	17.5	29.2	32.3	21.1	0.0062
	triple negative	127	9.4	36.2	40.9	13.4	
Clear cell renal cell carcinoma	ISUP 1	255	2.7	12.9	13.7	70.6	0.0066
	ISUP 2	374	4.5	11.5	16.8	67.1	
	ISUP 3	245	1.6	13.5	24.5	60.4	
	ISUP 4	68	0.0	17.6	25.0	57.4	
	Fuhrman 1	60	3.3	5.0	15.0	76.7	0.0076
	Fuhrman 2	643	3.1	12.0	17.0	68.0	
	Fuhrman 3	276	1.8	12.7	24.3	61.2	
	Fuhrman 4	82	0.0	20.7	22.0	57.3	
	Thoenes 1	332	2.1	12.0	16.0	69.9	0.0056
	Thoenes 2	462	3.7	14.1	20.3	61.9	
	Thoenes 3	89	0.0	15.7	30.3	53.9	
	UICC 1	298	2.3	9.4	14.4	73.8	<0.0001
	UICC 2	35	0.0	14.3	28.6	57.1	
	UICC 3	88	5.7	14.8	21.6	58.0	
	UICC 4	68	4.4	22.1	33.8	39.7	
	pT1	626	2.4	8.9	16.1	72.5	<0.0001
	pT2	129	0.8	16.3	22.5	60.5	
	pT3-4	311	3.9	17.7	23.8	54.7	
	pN0	165	2.4	10.9	23.0	63.6	0.2419
	pN+	23	8.7	21.7	17.4	52.2	
	pM0	104	4.8	9.6	16.3	69.2	0.0081
	pM+	86	3.5	19.8	30.2	46.5	
Papillary renal cell carcinoma	ISUP 1	34	0.0	17.6	32.4	50.0	0.8155
	ISUP 2	118	4.2	16.1	22.9	56.8	
	ISUP 3	73	1.4	15.1	23.3	60.3	
	ISUP 4	7	0.0	14.3	28.6	57.1	
	Fuhrman 1	2	0.0	50.0	0.0	50.0	0.4008
	Fuhrman 2	161	4.3	14.9	23.6	57.1	
	Fuhrman 3	75	0.0	13.3	24.0	62.7	
	Fuhrman 4	11	0.0	9.1	36.4	54.5	
	Thoenes 1	47	0.0	12.8	29.8	57.4	0.5469
	Thoenes 2	138	4.3	15.9	24.6	55.1	
	Thoenes 3	16	6.3	12.5	18.8	62.5	
	UICC 1	88	1.1	15.9	25.0	58.0	0.9823
	UICC 2	12	0.0	16.7	33.3	50.0	
	UICC 3	5	0.0	20.0	20.0	60.0	
	UICC 4	9	0.0	33.3	22.2	44.4	
	pT1	185	2.7	11.9	24.9	60.5	0.0153
	pT2	44	4.5	18.2	29.5	47.7	
	pT3-4	30	0.0	23.2	3.3	73.3	
	pN0	23	0.0	13.0	17.4	69.6	0.8002
	pN+	12	0.0	16.7	25.0	58.3	
	pM0	25	0.0	32.0	12.0	56.0	0.3955
	pM+	9	0.0	11.1	22.2	66.7	
Urothelial bladder carcinoma	pTa G2 low	345	5.8	7.8	12.5	73.9	<0.0001
	pTa G2 high	148	21.6	9.5	13.5	55.4	
	pTa G3	86	45.3	17.4	10.5	26.7	
	pT2	312	22.4	11.2	5.8	60.6	0.1904
	pT3	414	19.1	11.6	10.9	58.5	
	pT4	201	23.9	13.4	8.0	54.7	
	G2	76	22.4	10.5	9.2	57.9	0.8354 *
	G3	835	21.2	12.1	8.4	58.3	
	pN0	444	21.2	11.0	7.2	60.6	0.0051 *
	pN+	309	25.2	16.5	10.4	47.9	
	L0	162	25.9	10.5	6.8	56.8	0.0854 *
	L1	173	30.1	13.9	12.1	43.9	
	V0	276	24.3	11.2	10.5	54.0	0.9963 *
	V1	100	24.0	12.0	10.0	54.0	
	UICC 1-2	11	27.3	18.2	9.1	45.5	0.0466
	UICC 3	43	4.7	9.3	11.6	74.4	
	UICC 4	40	25.0	17.5	15.0	42.5	
Adenocarcinoma of the pancreas	pT1	8	0.0	0.0	37.5	62.5	0.3759
	pT2	49	2.0	18.4	42.9	36.7	
	pT3	295	0.0	13.2	46.8	40.0	
	pT4	23	0.0	8.7	39.1	52.5	
	G1	11	0.0	18.2	45.5	36.4	0.9853
	G2	266	0.4	13.2	45.9	40.6	
	G3	80	0.0	12.5	48.8	38.8	
	pN0	73	0.0	17.8	46.6	35.6	0.5177
	pN+	301	0.3	12.3	45.5	41.9	
Adenocarcinoma of the stomach	diffuse	65	52.3	35.4	9.2	3.1	0.1204
	inestinal	82	46.3	29.3	20.7	3.7	
	mixed	51	33.3	31.4	29.4	5.9	
	pN0	65	55.4	27.7	12.3	4.6	0.4248
	pN+	199	44.7	32.7	18.6	4.0	
	MMR proficient	229	41.0	34.1	20.5	4.4	0.0051
	MMR deficient	37	64.9	13.5	10.8	10.8	
Endometrioid endometrial carcinoma	pT1	105	79.0	16.2	3.8	1.0	0.2532
	pT2	23	65.2	34.8	0.0	0.0	
	pT3-4	36	72.2	16.7	8.3	2.8	
	pN0	50	66.0	30.0	4.0	0.0	0.0815
	pN+	30	73.3	13.3	6.7	6.7	
Serous carcinoma of the ovary	pT1	29	0.0	17.2	58.6	24.1	0.0720
	pT2	36	8.3	22.2	55.6	13.9	
	pT3	231	4.8	23.8	39.8	31.6	
	pN0	76	6.6	23.7	51.3	18.4	0.1145
	pN1	145	5.5	24.1	37.9	32.4	
Germ cell tumors of the testis	pT1	337	33.2	59.3	7.1	0.3	0.4978
	pT2	135	38.5	57.0	4.4	0.0	
	pT3-4	52	40.4	57.7	1.9	0.0	
	V0	433	32.3	60.7	6.7	0.2	0.0566
	V1	55	49.1	49.1	1.8	0.0	
	L0	379	33.0	60.2	6.6	0.3	0.4164
	L1	115	40.0	55.7	4.3	0.0	
	spermatic cord invasion	410	34.1	59.5	6.1	0.2	0.7932
	no spermatic cord invasion	56	37.5	58.9	3.6	0.0	
	rete testis invasion	234	30.8	62.0	7.3	0.0	0.1691
	no rete testis invasion	262	37.8	57.3	4.6	0.4	
	pM0	527	35.3	58.6	5.9	0.2	0.1508
	pM+	5	0.0	100.0	0.0	0.0	
Papillary carcinoma of the thyroid	pT1	136	0.0	3.7	40.4	55.9	0.453
	pT2	65	0.0	4.6	33.8	61.5	
	pT3-4	90	0.0	6.7	45.6	47.8	
	pN0	79	0.0	3.8	49.4	46.8	0.2827
	pN+	104	0.0	7.7	39.4	52.9	
Adenocarcinoma of the colorectum	pT1	79	81.0	16.5	2.5	0.0	0.3074
	pT2	415	82.2	14.5	2.4	1.0	
	pT3	1213	83.3	14.7	2.0	0.1	
	pT4	423	83.9	13.5	2.6	0.0	
	pN0	1117	82.3	15.6	2.1	0.1	0.2084
	pN+	1008	83.7	13.4	2.5	0.4	
	V0	1537	82.5	15.0	2.2	0.3	0.2568
	V1	556	84.2	13.1	2.7	0.0	
	L0	692	82.5	15.2	2.0	0.3	0.8962
	L1	1414	83.0	13.1	2.4	0.2	
	right side	440	86.6	11.6	1.8	0.0	0.552
	left side	1190	84.5	13.5	2.0	0.0	
	MMR proficient	1119	85.0	13.2	1.8	0.0	0.2494
	MMR deficient	82	82.9	12.2	4.9	0.0	
	RAS wildtype	453	83.7	14.3	2.0	0.0	0.3056
	RAS mutation	351	86.8	12.0	1.1	0.3	
	BRAF wildtype	121	83.5	14.9	1.7	0.0	0.5259
	BRAF V600E mutation	22	90.9	9.1	0.0	0.0	

* Only in pT2-4 urothelial bladder carcinomas. Abbreviations: pT: pathological tumor stage, G: grade, pN: pathological lymph node status, pM: pathological status of distant metastasis, V: venous invasion, L: lymphatic invasion, PR: progesterone receptor, MMR: mismatch repair, ER: estrogen receptor, ISUP: International Society of Urological Pathology, UICC: Union for International Cancer Control.

## Data Availability

All data generated or analyzed during this study are included in this published article.

## Supplementary Materials

The following supporting information can be downloaded at: https://www.mdpi.com/article/10.3390/biomedicines13071683/s1, Figure S1: IHC validation by comparison of two antibodies; Figure S2: IHC validation by comparison of two antibodies Table S1: List of studies used to generate Supplementary Figure S2.
