# Supplementary material for: Glucocorticoid Receptor (GR) Expression in Human Tumors: A Tissue Microarray Study on More than 14,000 Tumors
Source: Biomedicines. 2025 Jul 9;13(7):1683. doi: 10.3390/biomedicines13071683 (PMC12292884; doi:10.3390/biomedicines13071683)

Supplementary Figure 1. IHC validation by comparison of two antibodies. The panels show a concordance of immunostaining results obtained by two independent AR antibodies (HMV304, EPR19621). Using HMV-304, a moderate to strong nuclear GR positivity was seen in myoepithelial and luminal cells of the breast (A) and in throphoblastic cells of the first trimenon placenta (B). A reduced or absent GR staining was seen in crypt base epithelial cells of the small intestine (C), cells of the spermiogenesis (D), throphoblastic cells of the mature placenta (E), upper cell layers of non-keratinizing squamous epithelium (F) and urothelium (G) as well as in adrenocortical cells (H). Using EPR19621 a staining of identical cell types was seen in breast (I), first trimenon placenta (K), ileum (L), testis (M), mature placenta (N), squamous epithelium (O), urothelium (P) and the adrenal cortex (Q). The images A-H and I-Q are from consecutive tissue sections.

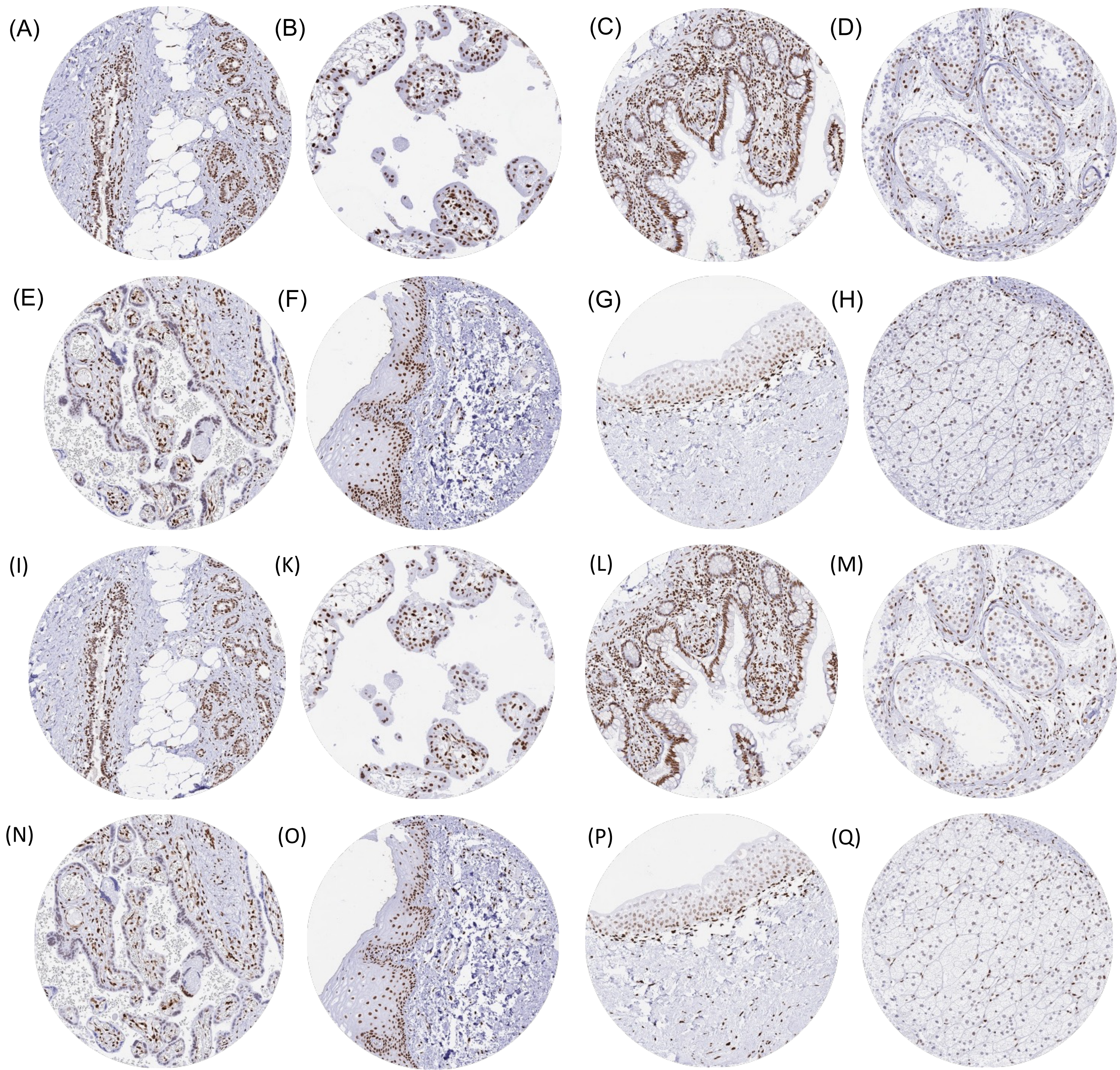

Supplement: Supplementary file 1 [file biomedicines-13-01683-s001.zip › Supplement Figure 1.pdf]
