# Supplementary material for: Glucocorticoid Receptor (GR) Expression in Human Tumors: A Tissue Microarray Study on More than 14,000 Tumors
Source: Biomedicines. 2025 Jul 9;13(7):1683. doi: 10.3390/biomedicines13071683 (PMC12292884; doi:10.3390/biomedicines13071683)
Supplement: Supplementary file 1 [file biomedicines-13-01683-s001.zip › Supplement Table 1_GR.pdf]

author, year	PMID	entity	analyzable tumors (n)	positive (%)
Amal Melhem et al., 2009	19383827	Clear cell carcinoma of the ovary	1	100,00%
Amal Melhem et al., 2009	19383827	Brenner tumor	1	100,00%
H-C Lien et al., 2006	16639692	Tubular carcinoma of the breast	2	0,00%
Ulrich Kaiser et al., 1996	8707409	Lung, neuroendocrine tumor (NET)	2	100,00%
Hitoshi Ishiguro et al., 2014	25015855	Squamous cell carcinoma of the bladder	3	66,67%
H-C Lien et al., 2008	18269582	Adenocarcinoma of the esophagus	4	0,00%
Zheng et al., 2012	23033490	muscle invasive urothelial carcinoma of the bla	5	100,00%
Amal Melhem et al., 2009	19383827	Serous carcinoma of the ovary	5	100,00%
Jennifer Taylor Veneris et al., 2017	28456378	Carcinosarcoma of the ovary	7	57,10%
H-C Lien et al., 2006	16639692	Mucinous carcinoma of the breast	8	0,00%
Thaddeus S Block et al., 2017	28293120	Mesothelioma, not otherwise classified	8	100,00%
Rita Nanda et al., 2016	27386391	ıvasive breast carcinoma, not otherwise classifie	9	66,67%
Thaddeus S Block et al., 2017	28293120	ıvasive breast carcinoma, not otherwise classifie	10	100,00%
Thaddeus S Block et al., 2017	28293120	Gallbladder adenocarcinoma	10	100,00%
Thaddeus S Block et al., 2017	28293120	Hepatocellular carcinoma	10	100,00%
Thaddeus S Block et al., 2017	28293120	Adenocarcinoma of the prostate	11	100,00%
Thaddeus S Block et al., 2017	28293120	Malignant melanoma	11	100,00%
Satoko Kakiuchi-Kiyota et al., 2014	23702648	Squamous cell carcinoma of the lung	11	54,50%
Evgeny Yakirevich et al., 2011	21531004	Oncocytoma	14	14,00%
Thaddeus S Block et al., 2017	28293120	Cervical carcinoma, not otherwise classified	15	100,00%
Evgeny Yakirevich et al., 2011	21531004	Chromophobe renal cell carcinoma	16	6,00%
Nishimura Kazua et al., 2001	11717335	Adenocarcinoma of the prostate	16	50,00%
Thaddeus S Block et al., 2017	28293120	Ductal adenocarcinoma of the pancreas	16	100,00%
Simone Lai et al., 2013	23072594	Malignant melanoma lymph node metastasis	17	41,18%
Ulrich Kaiser et al., 1996	8707409	Adenocarcinoma of the lung	17	88,24%
Satoko Kakiuchi-Kiyota et al., 2014	23702648	Adenocarcinoma of the lung	17	64,70%
Frederic Buxant et al., 2009	19697616	Squamous cell carcinoma of the cervix	17	100,00%
Ulrich Kaiser et al., 1996	8707409	Squamous cell carcinoma of the lung	17	94,12%
H-C Lien et al., 2006	16639692	Lobular carcinoma of the breast	18	0,00%
Yichun Zheng et al., 2012	23033490	uscle invasive urothelial carcinoma of the bladd	19	100,00%
Rezvan Abduljabbar et al., 2015	25762479	Medullary carcinoma of the breast	20	40,00%
James. L. Mohler et al., 1996	9816246	Adenocarcinoma of the prostate	20	20,00%
H-C Lien et al., 2008	18269582	Gallbladder adenocarcinoma	20	20,00%
James Norman et al., 1994	7518883	Ductal adenocarcinoma of the pancreas	20	60,00%
Congcong Xu et al., 2020	32850423	muscle invasive urothelial carcinoma of the bla	21	90,50%
H-C Lien et al., 2008	18269582	Pancreatic/Ampullary adenocarcinoma	21	19,05%
Yen-Shen Lu et al., 2006	16806572	Squamous cell carcinoma of the lung	21	61,90%
Evgeny Yakirevich et al., 2011	21531004	Papillary renal cell carcinoma	23	26,00%
Wenjun Chang et al., 2009	19569046	Gastric Adenocarcinoma, not otherwise classifie	26	42,31%
Erica M. Stringer-Reasor et al., 2015	26115975	Serous carcinoma of the ovary	27	92,59%
Congcong Xu et al., 2020	32850423	uscle invasive urothelial carcinoma of the bladde	28	75,00%
Ingvild L. Tangen et al., 2017	28927900	Endometrial clear cell carcinoma	28	64,00%
Ingvild L. Tangen et al., 2017	28927900	Carcinosarcoma of the uterus	28	75,00%
H-C Lien et al., 2008	18269582	Hepatocellular carcinoma	28	92,86%
Umaima Al-Alem et al., 2021	34068181	Lobular carcinoma of the breast	29	48,00%
Jennifer Taylor Veneris et al., 2017	28456378	Mucinous carcinoma of the ovary	30	13,33%
Isabel Conde et al., 2008	17952860	Lobular carcinoma of the breast	34	29,40%
H-C Lien et al., 2008	18269582	Adenocarcinoma of the colon	35	0,00%
Frederic Buxant et al., 2010	19875955	ıvasive breast carcinoma, not otherwise classifie	39	43,59%
H-C Lien et al., 2008	18269582	Ductal adenocarcinoma of the pancreas	41	26,83%
Luana Samara Balduíno de Sena et al.	29935090	Squamous cell carcinoma of the skin	44	100,00%
H-C Lien et al., 2006	16639692	Phyllodes tumour of the breast	44	50,00%
Eiji Kashiwagi et al., 2016	27635763	Urothelial carcinoma of the kidney pelvis	45	57,78%
Yen-Shen Lu et al., 2005	16419168	ıvasive breast carcinoma, not otherwise classifie	45	11,10%
Isabel Conde et al., 2008	17952860	Invasive breast carcinoma of no special type	46	39,10%
Jennifer Taylor Veneris et al., 2017	28456378	Endometrioid carcinoma of the ovary	47	10,64%
Gabrielle M Baker et al., 2015	26673410	ıvasive breast carcinoma, not otherwise classifie	49	84,00%
Jennifer Taylor Veneris et al., 2017	28456378	Clear cell carcinoma of the ovary	50	26,00%
H-C Lien et al., 2008	18269582	Cholangiocarcinoma	50	28,00%
Lyndal J Tacon et al., 2009	19820023	Adrenal cortical carcinoma	51	88,24%
Raja Alyusuf et al., 2017	29348941	Invasive breast carcinoma of no special type	52	98,00%
H-C Lien et al., 2008	18269582	Squamous cell carcinoma of the esophagus	52	98,10%
H-C Lien et al., 2008	18269582	Gastric Adenocarcinoma, not otherwise classifie	54	7,41%
Yen-Shen Lu et al., 2006	16806572	Adenocarcinoma of the lung	55	45,45%
Xavier Catteau et al., 2016	27699028	ıvasive breast carcinoma, not otherwise classifie	56	53,60%
Simone Lai et al., 2013	23072594	Malignant melanoma	64	35,94%
Hitoshi Ishiguro et al., 2014	25015855	uscle invasive urothelial carcinoma of the bladde	65	73,84%
Ingvild L. Tangen et al., 2017	28927900	Endometrial serous carcinoma	67	61,00%
Yoshitsugu Mitani et al., 2020	31772120	Salivary duct carcinoma	67	62,69%
Mugdha Patki et al., 2018	30375484	Adenocarcinoma of the lung	70	78,57%
Mingzhen Cai et al., 2022	35103835	Invasive breast carcinoma of no special type	72	97,00%
Lyndal J Tacon et al., 2009	19820023	Adrenal cortical adenoma	74	2,70%
Kan Wu et al., 2022	35992138	Adrenal cortical carcinoma	78	90,00%
Rezvan Abduljabbar et al., 2015	25762479	Lobular carcinoma of the breast	80	85,00%
Hitoshi Ishiguro et al., 2014	25015855	muscle invasive urothelial carcinoma of the bla	84	96,43%
Stamatios Theocharis et al., 2003	14560994	Adenocarcinoma of the colon	91	48,00%
Shunsuke Ueki et al., 2020	32106831	Squamous cell carcinoma of the esophagus	98	53,10%
Yalan Deng et al., 2021	34873175	Ductal adenocarcinoma of the pancreas	101	69,31%
Takahiro Mimae et al., 2011	21437890	Thymoma	103	85,44%
Takanobu Sasaki et al., 2023	37351994	Adenocarcinoma of the lung	116	66,00%
Zhongbo Han et al., 2021	34017210	Adenocarcinoma of the colon	122	46,72%
Russell Z Szmulewitz et al., 2012	21563193	Adenocarcinoma of the prostate	126	38,00%
Ximena Garcia et al., 2019	31950383	ıvasive breast carcinoma, not otherwise classifie	127	49,00%
Ayako Kanai et al., 2020	31989378	Invasive breast carcinoma of no special type	131	60,30%
Keely M Mc Namara et al., 2018	29563635	ıvasive breast carcinoma, not otherwise classifie	139	51,00%
Evgeny Yakirevich et al., 2011	21531004	Clear cell renal cell carcinoma	147	66,00%
Anniina Jääskeläinen et al., 2019	31704812	ıvasive breast carcinoma, not otherwise classifie	152	92,10%
J.S. Prabhu et al., 2021	34220400	ıvasive breast carcinoma, not otherwise classifie	175	74,00%
Umaima Al-Alem et al., 2021	34068181	Invasive breast carcinoma of no special type	191	42,00%
H-C Lien et al., 2006	16639692	Invasive breast carcinoma of no special type	223	2,24%
Larissa Belova et al., 2009	18668364	Invasive breast carcinoma of no special type	227	18,00%
Bernd Peter Kost et al., 2019	30306311	Cervical carcinoma, not otherwise classified	250	92,40%
Jennifer Taylor Veneris et al., 2017	28456378	Serous carcinoma of the ovary	265	48,30%
Ingvild L. Tangen et al., 2017	28927900	Endometrioid endometrial carcinoma	582	16,00%
Rezvan Abduljabbar et al., 2015	25762479	Invasive breast carcinoma of no special type	846	59,50%
Alexander Yemelyanov et al., 2007	17016446	Adenocarcinoma of the prostate	116	24,00%
