# Supplementary material for: Glucocorticoid Receptor (GR) Expression in Human Tumors: A Tissue Microarray Study on More than 14,000 Tumors
Source: Biomedicines. 2025 Jul 9;13(7):1683. doi: 10.3390/biomedicines13071683 (PMC12292884; doi:10.3390/biomedicines13071683)

Supplementary Figure 2. Graphical representation of GR data from this study (marked with a cross) in comparison with data from existing literature (marked with dots). In order to simplify the figure, the percentage of weak, moderate and strong staining was merged. Red dots are used for previous studies involving 1-20 cases, yellow dots for studies involving 20-100 cases and green dots for studies involving > 100 cases. All studies are quoted in a list of references in S1 Table.

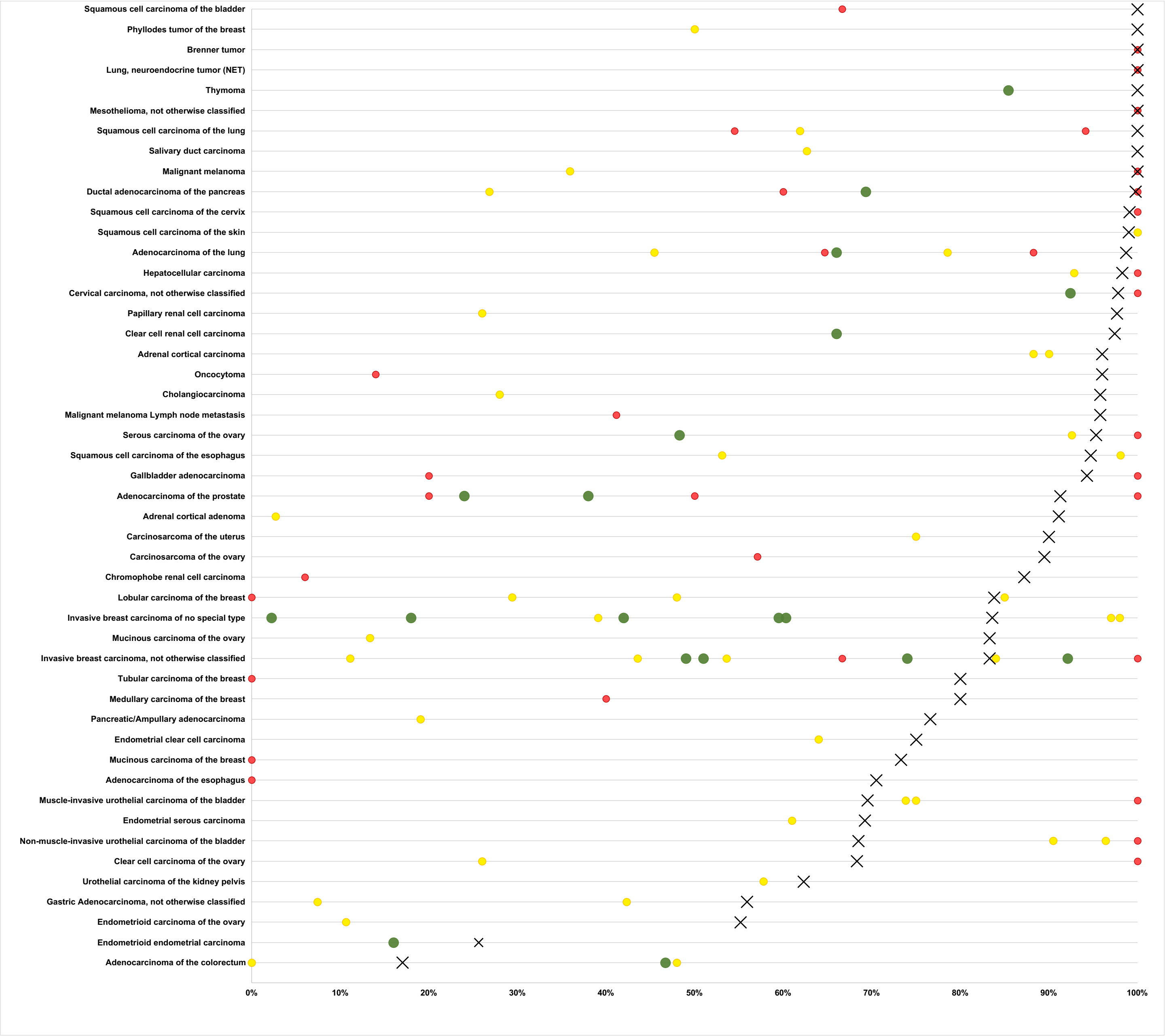

Supplement: Supplementary file 1 [file biomedicines-13-01683-s001.zip › Supplementary Figure 2.pdf]
